# Influence of DNA-Polymorphisms in Selected Circadian Clock Genes on Clock Gene Expression in Subjects from the General Population and Their Association with Sleep Duration

**DOI:** 10.3390/medicina58091294

**Published:** 2022-09-16

**Authors:** Rocío Barragán, José V. Sorlí, Oscar Coltell, Inmaculada Gonzalez-Monje, Rebeca Fernández-Carrión, Laura V. Villamil, Olga Portolés, Dolores Corella, Carolina Ortega-Azorín, Eva M. Asensio

**Affiliations:** 1 Department of Preventive Medicine and Public Health, School of Medicine, University of Valencia, 46010 Valencia, Spain; 2 CIBER Fisiopatología de la Obesidad y Nutrición, Instituto de Salud Carlos III, 28029 Madrid, Spain; 3 Department of Computer Languages and Systems, Universitat Jaume I, 12071 Castellón, Spain; 4 Department of Physiology, School of Medicine, University Antonio Nariño, 111511 Bogotá, Colombia

**Keywords:** clock genes, gene expression, sex differences, sleep traits

## Abstract

*Background and Objectives*: Circadian rhythms have an important implication in numerous physiological and metabolic processes, including the sleep/wake cycle. Inter-individual differences in factors associated with circadian system may be due to gene differences in gene expression. Although several studies have analyzed the association between DNA polymorphisms and circadian variables, the influence on gene expression has been poorly analyzed. Our goal was to analyze the association of genetic variations in the clock genes and the gene expression level. *Materials and Methods*: We carried out a cross-sectional study of 102 adults (50.9% women). RNA and DNA were isolated from blood and single-nucleotide polymorphisms (SNPs), and the main circadian clock genes were determined. Gene expression of *CLOCK*, *PER1*, and *VRK2* genes was measured by Reverse-transcription polymerase chain reaction (RT-PCR). The association between the DNA-SNPs and gene expression was analyzed at the gene level. In addition, a polygenic risk score (PRS), including all the significant SNPs related to gene expression, was created for each gene. Multivariable model analysis was performed. *Results*: Sex-specific differences were detected in *PER1* expression, with these being higher in women (*p* = 0.034). No significant differences were detected in clock genes expression and lifestyle variables. We observed a significant association between the *ARNTL*-rs7924734, *ARNTL*-rs10832027, *VRK2*- rs2678902 SNPs, and *CLOCK* gene expression; the *PER3*-rs228642 and *PER3*-rs10127838 were related to *PER1* expression, and the *ARNTL*-rs10832027, *ARNTL*-rs11022778, and *MNTR1B*-rs10830963 were associated with *VRK2* gene expression (*p* < 0.05). The specific PRS created was significantly associated with each of the gene expressions analyzed (*p* < 0.001). Finally, sleep duration was associated with *PER3*-rs238666 (*p* = 0.008) and *CLOCK*-rs4580704 (*p* = 0.023). *Conclusion*: We detected significant associations between DNA-SNPs in the clock genes and their gene expression level in leukocytes and observed some differences in gene expression per sex. Moreover, we reported for the first time an association between clock gene polymorphisms and *CLOCK*, *PER1*, and *VRK2* gene expression. These findings need further investigation.

## 1. Introduction

The circadian timing system consists of the brain’s central clock in the hypothalamic suprachiasmatic nucleus (SCN) and various clocks in peripheral tissues, including muscle, adipose tissue, and liver, that orchestrate 24-hour cycles at the transcriptional level [1,2]. The SCN receives a 24-hours solar day via a pathway from the retina that synchronize the circadian rhythm of SCN [2]. The circadian clock is implicated in the regulation of numerous cellular, physiological, metabolic, endocrinal, and behavioral systems. The circadian system regulates energy expenditure, food intake, body insulin sensitivity, and the sleep–wake cycle [3,4].

In mammals, the central circadian rhythm is regulated by an autonomous transcription–translation feedback loop of cells that provides a pattern of rhythmic expression to the clock genes. During the day, the Circadian Locomotor Output Cycles Kaput (*CLOCK*) transcription factors and Neuronal PAS Domain Protein 2 (*NPAS2*) form complexes with Brain and Muscle ARNT-Like 1-2 (*ARNTL* or *BMAL1* and *ARNTL2* or *BMAL2*) in order to activate the transcription of Period Circadian Clock (*PER1/PER2*) and Cryptochrome Circadian Clock (*CRY1/CRY2*). The heterodimerized proteins Period Circadian Regulator (PER) and Cryptochrome Circadian Regulator (CRY) move to the nucleus and suppress the action of *CLOCK/BMAL1*, thus forming a negative feedback loop [5]. Moreover, recent genome-wide association studies GWASs have associated the Vaccinia-related Kinase (VRK) Serine/Threonine Kinase 2 *(VRK2*) gene with the circadian cycle and, more specifically, with sleep [6,7] although the mechanism has yet to be explained.

It has, however, been shown that alterations in lifestyle patterns due to social or occupational patterns can contribute to the disruption of the circadian cycle [1,4], with possible effect on metabolic risk factors [8]. Furthermore, genetic differences may also be associated with inter-individual factors associated with the circadian system [9]. Thus, gene expression analysis may be a potential method for identifying molecular pathways associated with cardiometabolic diseases [10,11]. Our group is well-acquainted with the importance of genes involved in the circadian cycle, having observed a significant association between the *CLOCK*-rs4580704 single-nucleotide polymorphism (SNP) and type 2 diabetes (T2D) incidence in 3671 non-T2D Prevention with Mediterranean Diet (PREDIMED) study participants, suggesting a gene effect modulation induced by the Mediterranean diet [3]. In addition, we recently observed that the association between genetic variations in the melatonin receptor 1B gene (*MTNR1B*) consisting of the rs10830963 SNP was age-dependent, having an increased effect in young populations in comparison with older populations [12]. However, as far as we know, very few studies have analyzed the association between gene expression and clock DNA polymorphisms or sleep-related variables in humans. Therefore, our aim was to analyze the effect of selected DNA-SNPs in the main clock genes on leukocyte gene expression. In addition, we analyzed the modulation by sex and other lifestyle variables as well as their association with sleep-related variables.

## 2. Materials and Methods

### 2.1. Study Subjects

We carried out a cross-sectional study in a subsample of subjects from a general Mediterranean population participating in the Obesity, Nutrition & Information and Communication Technologies (OBENUTIC) study. Briefly, OBENUTIC is a case-controlled study of more than 1000 Caucasian participants aged between 18 and 80 years old from the Valencia Region, Spain. Inclusion criteria have been described previously [13]. We randomly selected 130 participants for the clock gene expression sub-study. Finally, we included a total of 102 participants for gene expression analysis after discarding those that did not meet RNA quality. The study was undertaken at the Department of Preventive Medicine and Public Health, School of Medicine at the University of Valencia, Valencia. Participants provided written informed consent, and study protocol and procedures were approved according to the ethical standards of the Helsinki Declaration and by the Human Research Ethics Committee of the University of Valencia, Valencia (reference number: H1488282121722; 6 April 2017).

### 2.2. Lifestyle and Clinical Evaluation

Socio-demographic and lifestyle variables (smoking, diet, and physical activity) were obtained through a standardized questionnaire previously used in our studies. In addition, we gathered information on various weekday and weekend sleep-related items. Participants were asked: (1) How many hours do you sleep on weekdays? (2) How many hours do you sleep on Saturdays? (3) How many hours do you sleep on Sunday? In addition, we asked the same questions, but this time related to the time of awakening. From these data, we calculated the following variables: Average sleep duration (h) = ((5 × sleep duration on weekdays) + sleep duration on Saturdays + sleep duration on Sunday)/7; Average waketime= ((5 × awakening time on weekdays) + awakening time on Saturdays + awakening time on Sunday)/7. Average weekend waketime was calculated with the previous formula but using only Saturday and Sunday data.

Participants were measured for height with a standard stadiometer (SECA Mod 220, SECA Deutschland Gmbh & Co, Hamburg, Germany). Weight was determined by bioimpedance using a TANITA-BC-420-S scale (Tanita Corporation 1-14-2 Maeno-Cho, Habashi-ku, Tokyo, Japan). Waist circumference was measured at the midpoint between the lowest rib and the iliac crest after a normal exhalation (ISAK, 2011). Systolic and diastolic blood pressure was obtained using an automatic sphygmomanometer (OMRON HEM-705CP). Two blood pressure readings were taken in sitting position, resting 5 min between each measurement and considering the level of blood pressure as the mean of the readings. Blood samples were obtained after an overnight fast. Fasting glucose and lipids were measured using a Beckman Coulter Olympus AU5400 (Pasadena, CA, USA).

### 2.3. DNA Extraction and Genotyping

Genomic DNA was isolated from white blood cells. The *CLOCK*-rs4580704, *ARNTL*-rs1982350, and *MTNR1B*-rs10830963 were genotyped with an ABI Prism 7900HT Sequence Detection System (Applied Biosystems, Foster City, CA, USA) using a fluorescent allelic discrimination TaqManTM assay. Genotype frequencies did not deviate from Hardy–Weinberg equilibrium expectations.

Besides the genotyping of these pre-selected polymorphisms in the clock genes, a high-density genotyping at the genome-wide level using the Illumina Infinium OmniExpress-24 BeadChip genotyping array (ver. 1.0 and 1.1) or the Illumina Global Screening Array (GSA) (ver. 1.0) (Illumina Inc., San Diego, CA, USA), was then carried out as previously described [14]. Allele detection and genotype calling were performed in the GenomeStudio genotyping module (ver. 2.0.5) (Illumina, Inc., San Diego, CA, USA), and quality-control procedures were applied. We then selected the main clock genes (*CLOCK*, *ARNTL1*, *PER1*, *PER3*, and *VRK2*) and extracted all the genotyped SNPs in these genes using a dedicated Python script. As the SNP coverage of the OmniExpress-24 array is different from that of the GSA array, we only analyzed SNPs that matched in both arrays in order to only use directly genotyped SNPs instead of the imputed ones. We tested the linkage disequilibrium between the available SNPs in the clock genes, and only independent SNPs were used in further analyses.

### 2.4. RNA Isolation, cDNA Synthesis, and qPCR

Total RNA was extracted from plasma samples using Trizol Reagent, employing the manual method described by Chomczynski P and Sacchi N in 1987 [15]. RNA was quantified by measuring absorbance at 260 and 280 nm using a NanoPhotometer^TM^ spectrophotometer Implen P300 (Implen, Munich, Germany), and integrity was checked using an Agilent 2100 Bioanalyzer (Agilent Technologies, Foster City, CA, USA). Briefly, 500 ng total RNA from all samples were reverse transcribed using the High-Capacity cDNA Reverse Transcription Kit (Applied Biosystem, Foster City, CA, USA) and GeneAmp Polymerase Chain Reaction PCR System 9700 RT-PCR (Applied Biosystem, Foster City, CA, USA). Quantitative real-time PCR was performed using an ABI PRISM 7900 HT Sequence Detection System (Applied Biosystem). The genes were amplified using commercially available Taqman probes (Applied Biosystems). The Taqman Gene Expression kit was used for real-time PCR analysis. Duplicate amplification reactions were performed under the following conditions: 50 °C for 2 min, 95 °C for 10 min, and 40 cycles of 95° for 15 s and 60 °C for 1 min. The software used for the analysis of the results was Sequence Detection Systems (SDS) Automation Controller Software ver. 2.3 and Relative Quantitation (RQ) manager ver. 1.2 (Applied Biosystem, Foster City, CA, USA). The relative mRNA level for each transcript was calculated by the methods proposed by Livak KJ et al. for gene expression. Gene expression values at baseline are shown as 2-ΔCt [10,16]. 

### 2.5. Statistical Analysis

Anthropometric, blood pressure, and biochemical measurements were expressed as mean ± SD (standard deviation). The results for genes expression are presented as mean ± SEM (standard error of mean). Variables that did not fulfil normal distribution criteria were Ln transformed to improve symmetry for subsequent analyses. A polygenic risk score (PRS) for each clock gene was created by the unweighted sum of the highly significant clock SNP related with circadian phenotypes. Individual participant scores were created by adding up the number of risk alleles at each SNP. To test the differences between clock genes expression and lifestyle or clinical variables, we used linear regression tests. Linear regression coefficients were used to analyze the associations between SNPs genotypes and genes expression. Multivariate models were carried out to adjust for confounding factors. Statistical analyses were performed with IBM SPSS Statistics, version 27.0 (Armonk, NY, USA). All tests were two-tailed, and *p*-values < 0.05 were considered statistically significant.

## 3. Results

### 3.1. Population Characteristics

The 102 participants included in the analysis, 52 being women, had an average age of 43.1 ± 13.8 years and had a Body mass index (BMI) of 27.2 ± 5.1 kg/m^2^. Clinical and lifestyle characteristics are represented in Table 1. Briefly, 30.4% of the whole population presented obesity, 25.7% were hypertensive, 16.5% were current smokers, 33% had hypercholesterolemia, and 5% had diabetes. Differences per sex were detected in the prevalence of hypertension, obesity, and diabetes, which were higher in men (*p* ˂ 0.05). Individual clinical parameters also showed the same differences between males and females (Table 1).

### 3.2. Circadian Clock Gene Expression

**Figure 1:**
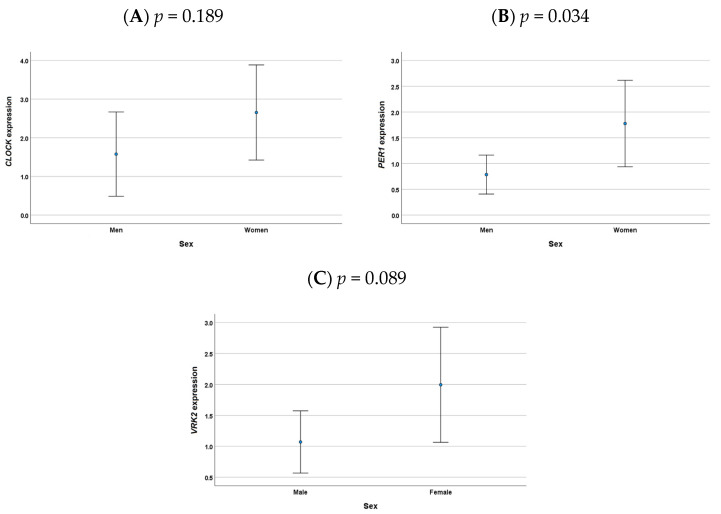
Representation of the clock gene expression by sex. (**A**) Differences in *CLOCK* genes between males and females were not statistical significance (*p* = 0.189). (**B**) Females showed higher *PER1* gene expression than men (*p* = 0.034). (**C**) A trend was observed for a higher *VRK2* gene expression in females than in males (*p* = 0.089).

Results of the global peripheral blood expression genes are represented in Figure 1. Women showed higher clock genes expression than men in all the genes analyzed, but this difference was only significant for *PER1* expression (*p* = 0.034).

No differences in these gene expressions were found according to age, obesity, diabetes, diet, sleep (duration and waketime), or smoking. However, in this last lifestyle factor, we detected a lower *VRK2* expression in smokers, but it did not reach statistical significance (β: −1.30 ± 0.70; *p* = 0.067), Table S1.

### 3.3. Impact of the Selected SNPs on Clock Gene Expression

We analyzed the differences in clock genes expression in all clock genes using the Infinium Ommni Express Array and the Infinium Global Screening Array methods. This netted a total of 32 polymorphic sites in the *CLOCK*, *ARNTL*, *PER1*, *PER3*, and *VRK2* genes. We also analyzed 3 more TaqIB SNP in the *CLOCK*, *ARNTL*, and *MTNR1B* genes, with a total of 35 SNPs analyzed. Of these 35 polymorphisms, 1 was a duplicate (rs1982350), and a total of 7 SNPs did not reach the Hardy–Weinberg equilibrium expectations at *p* < 0.05, so we removed it from our analysis. Of these 28 polymorphisms, 4 polymorphisms (rs1982350, rs7924734, rs10832027, and rs2678902) were associated with *CLOCK* gene expression. Two more SNPs (rs228642 and rs10127838) in the *PER3* gene were associated with *PER1* expression, and for the *VRK2* gene, three polymorphisms were linked (rs10832027, rs11022778, and rs10830963) with its expression (Table 2).

Furthermore, we tested whether these polymorphisms were related to sleep factors, and we found an association between the rs238666 in the *PER3* gene (β: 0.354 ± 0.354, *p* = 0.008) and the rs4580704 in *CLOCK* gene (β: −0.360 ± 0.155, *p* = 0.023) with weekly sleep duration after adjusting for sex and age. Similar results were detected for the two SNPs in the *VRK2* gene and waketime on the weekends (rs1051061: β: 0.435 ± 0.187, *p* = 0.023; and rs848291: β: −0.437 ± 0.187, *p* = 0.022.

In addition, we created a specific PRS with the most relevant SNPs for each clock gene. Thus, we detected that the three PRS could be used as a predictor of clock genes expression: *CLOCK* expression = β: 0.516 ± 0.135, *p* < 0.001; *PER1* expression = β: 0.867 ± 0.248, *p* = 0.001, and *VRK2* expression = 0.508 ± 0.127, *p* < 0.001 (Table 2).

## 4. Discussion

We analyzed gene expression levels in leukocytes of the main clock-related genes in a Mediterranean general population. To our knowledge, this study provides the first evidence of sex-differences expression of core molecular clock components in human leukocytes from blood. We also found, for the first time, differences in clock gene expressions according to different genotypes from circadian genes (*ARNTL*, *PER3*, and *VRK2*). However, due to our sample size, these findings need further investigation in a larger population and in different cohorts.

There is strong evidence of sex differences in physiology, health, and diseases. These differences have been attributed to hormones, sex chromosomes, genotypes, behavior, or environmental exposures [17]. To date, the underlying mechanisms continue to be unknown. The Genotype-Tissue Expression (GTEx) project has evaluated sex gene regulation in 29 human healthy tissue using 8279 whole-genome expression profiles to understand the biological mechanism basis of sex differences [18]. They found that the number of genes differentially expressed by sex is small (around 64 genes) in some tissues apart from the breast [18]. Our sex-specific analyses suggest that females present a higher clock gene expression in all the genes analyzed. However, statistical significance was achieved only for the *PER1* gene. Similar results were detected by Gómez-Abellán in obese women in the expression of *PER2*, *ARNTL1*, and *CRY1* in adipose tissue [19]. Lim et al. found a timing expression of three clock genes (*PER2*, *PER3*, and *ARNTL1*) earlier in females than in men [20], and this also could explain the differences per sex identified in our sample. Our findings may confirm the growing evidence of sex differences in circadian rhythms, such as intrinsic period, chronotype, or differences in sleep phases [20,21], and according to some authors, estrogens could partly modulate circadian clock genes expression [22]. On the other hand, although we did not find any significant differences in clock gene expression by lifestyle and clinical variables, other authors detected that clock genes expression could be influenced by other factors such as sleep disturbances and chronic pathologies. Emekli et al. noticed a decrease in the expression levels of *PER1* and *PER2* genes in night shift workers and patients with chronic insomnia [23]. One week of insufficient sleep (5.70 h) was linked with the expression of circadian rhythms, mainly involving the *PER2* and *PER3* genes, two of the most down-regulated genes after sleep deprivation intervention [24]. Furthermore, studies in mice observed that 28 days of sleep restriction was associated with the disappearance of circadian rhythms in the *Cry1* and *Per1*/*2*/*3* genes [25]. Obesity [26] and diabetes [27,28] have been also related to a reduction in the expression of clock genes in peripheral leucocytes. Since these results raise more questions, future studies including epigenetic variability—essential in the gene regulatory process—are necessary.

There have been very few studies analyzing the gene expression of the main genes related to circadian rhythm and clock polymorphisms, with our results adding new information to the field. In particular, we showed that specific SNPs in the *ARNTL* (rs7924734, rs10832027, rs11022778), *PER3* (rs228642, rs10127838), *VRK2* (rs2678902), and *MTNR1B* (rs10830963) genes presented differences in their clock gene expression according to genotypes. Unexpectedly, we did not notice significant results in the *CLOCK*’s polymorphisms. A prior study in mice found that the rs1801260 resulted in differences in *CLOCK* and *PER2* expression according to genotype, with the ones that had a greater expression being the carriers of the mutated allele [29]. We could not observe this difference, probably due to our sample size and differences between humans and mice. Changes in the expression of circadian genes may have an implication on physiological processes, as the molecular clock core regulates hormonal, sleep/wake, food intake, or blood pressure rhythms. Thus, clock gene expression has been related to different components of cardiometabolic risk factors. Vieira et al. detected alterations in clock gene expression in visceral adipose from obese subjects and a correlation between *CLOCK* gene and large-density lipoprotein (LDL) levels [30]. Similar results were found by Gómez-Abellán in morbidly obese women. *PER2* expression was inversely correlated to waist circumference, and *ARNTL 1*, *PER1*, and *CRY1* were also negatively associated with LDL cholesterol and total cholesterol [31].

Genetic variations could explain some phenotypic consequence, such as duration of sleep, waketime, addiction, insomnia, day preferences, etc. In our work, we noticed that the polymorphisms rs238666 in the *PER3* and the rs4580704 in the *CLOCK* gene were associated with sleep duration and the rs1051061 and rs848291 in the *VRK2* with waketimes. Heritability of sleep duration has been estimated as 46% of the variability and 44% for sleep quality. However, these values vary substantially with age, being less influential at younger ages [32]. Moreover, in recent years, different GWAS studies related to sleep traits have been carried out [33,34,35]. These results highlight the role of genetic factors in explaining differences in sleep parameters among the population. Nevertheless, to validate this association, further characterization of the molecular consequences of these polymorphisms is required and evaluating clock gene expression could be one approach [36].

## 5. Conclusions

In short, in these Mediterranean subjects, we observed that some polymorphisms in the circadian rhythm genes are significantly associated with one or more of the main clock genes expressed in leukocytes from blood. Moreover, we detected sex-specific differences in gene expression levels of circadian genes. Changes in gene expression could be a measure in the development of metabolic disturbances and a biomarker for effective treatments of metabolic pathologies.

## Figures and Tables

**Table 1:** General characteristics of the study participants.

	Total (*n* = 102)	Male (*n* = 50)	Female (*n* = 52)	*p*
Age (years)	43.1 ± 13.8	44.0 ± 13.5	42.3 ± 14.2	0.531
BMI (kg/m^2^)	27.2 ± 5.1	29.0 ± 4.7	25.5 ± 4.8	˂0.001
Waist circumference (cm)	92.0 ± 16.2	100.5 ± 15.1	83.9 ± 12.6	˂0.001
SBP (mm Hg)	123.3 ± 16.4	129.1 ± 14.4	117.9 ± 16.5	˂0.001
DBP (mm Hg)	77.9 ± 10.5	80.5 ± 12.8	75.5 ± 7.2	0.020
Total cholesterol (mg/dL)	207.4 ± 40.1	206.0 ± 36.0	208.6 ± 43.9	0.745
LDL cholesterol (mg/dL)	136.1 ± 32.5	138.7 ± 30.2	133.6 ± 34.7	0.434
HDL cholesterol (mg/dL)	56.9 ± 14.1	49.0 ± 9.8	64.5 ± 13.4	˂0.001
Triglycerides (mg/dL)	106.7 ± 63.0	127.2 ± 71.6	87.3 ± 46.4	˂0.001
Fasting glucose (mg/dL)	95.1 ± 26.2	101.8 ± 34.8	88.7 ± 10.4	0.013
Hypertension (%)	25.7	38.8	13.5	0.004
Obesity cases (%)	30.4	42.0	19.2	0.012
Type 2 diabetes (%)	5	10.2	0.0	0.018
Hypercholesterolemia (%)	33	37.5	28.8	0.358
Current smoker (%)	16.5	13.3	19.2	0.435
Sedentary (%)	26.5	30.4	23.1	0.380
Sleep duration (h)	7.1 ± 0.9	7.1 ± 0.9	7.1 ± 0.9	0.791
Waketime (hour:min)	7:45 ± 1:02	7:48 ± 1:18	7:42 ± 0:42	0.573

Values are mean ± SE (standar error) for continuous variables and % for categorical variables; *p*, *p*-value for the comparisons (means or %) between men and women; SBP, systolic blood pressure; DBP, diastolic blood pressure; LDL, large-density lipoprotein; HDL, high-density lipoprotein obesity, BMI ≥ 30 kg/m^2^; type 2 diabetes, antidiabetic drug or glucose ≥ 126 mg/dL; hypercholesterolemia, hypolipidemic drugs or LDL cholesterol ≥ 160 mg/dL; hypertension, antihypertensive drug or SBP ≥ 140 mmHg or DBP ≥ 90 mmHg; Waketime was represented as hours:minute. BMI, body mass index.

**Table 2:** Relationship between clock gene polymorphisms and clock gene expression.

		*CLOCK* Gene Expression		*PER1* Gene Expression		*VRK2* Gene Expression	
SNP	Gene	β ± SE	*p*-value	β ± SE	*p*-value	β ± SE	*p*-value
rs4580704	*CLOCK*	0.296 ± 0.462	0.524	0.153 ± 0.468	0.745	0.221 ± 0.426	0.606
rs1056547	*CLOCK*	−0.112 ±0.434	0.797	−0.425 ± 0.436	0.333	−0.153 ± 0.406	0.702
rs1801260	*CLOCK*	−0.203 ± 0.443	0.648	−0.326 ± 0.440	0.461	−0.529 ± 0.403	0.193
rs1982350	*ARNTL*	−0.711 ± 0.414	0.090	−0.94 ± 0.441	0.831	−0.594 ± 0.385	0.126
rs10741616	*ARNTL*	−0.600 ± 0.442	0.179	0.316 ± 0.462	0.496	−0.303 ± 0.417	0.471
rs7924734	*ARNTL*	1.092 ± 0.484	0.027	0.259 ± 0.523	0.622	0.881 ± 0.455	0.056
rs6486121	*ARNTL*	0.513 ± 0.400	0.204	−0.353 ± 0.403	0.385	0.055 ± 0.375	0.883
rs10832027	*ARNTL*	0.959 ± 0.469	0.044	0.302 ± 0.506	0.552	1.043 ± 0.435	0.019
rs7937060	*ARNTL*	0.561 ± 0.390	0.155	−0.222 ± 0.396	0.557	0.073 ± 0.373	0.845
rs11022778	*ARNTL*	−0.756 ± 0.442	0.091	−0.623 ± 0.446	0.166	−1.193 ± 0.401	0.004
rs969485	*ARNTL*	−0.497 ± 0.447	0.270	−0.384 ± 0.447	0.393	−0.708 ± 0.411	0.089
rs2518023	*PER1*	0.749 ± 1.066	0.484	0.426 ± 1.085	0.696	0.741 ± 1.000	0.461
rs228729	*PER3*	0.196 ± 0.459	0.670	−0.149 ± 0.458	0.746	−0.182 ± 0.427	0.671
rs707467	*PER3*	0.617 ± 0.631	0.332	−0.436 ± 0.662	0.513	0.522 ± 0.596	0.384
rs228642	*PER3*	−0.392 ± 0.496	0.431	−1.180 ± 0.487	0.018	−0.364 ± 0.468	0.439
rs228666	*PER3*	−0.255 ± 0.406	0.532	0.152 ± 0.410	0.711	−0.492 ± 0.384	0.205
rs10127838	*PER3*	0.568 ± 0.427	0.188	0.922 ± 0.429	0.035	0.731 ± 0.398	0.071
rs2678902	*VRK2*	0.987 ± 0.434	0.026	0.728 ± 0.453	0.112	0.607 ± 0.417	0.150
rs2678880	*VRK2*	−0.093 ± 0.409	0.821	0.040 ± 0.415	0.923	−0.219 ± 0.387	0.574
rs7596775	*VRK2*	0.382 ± 0.410	0.355	−0.331 ± 0.417	0.430	−0.144 ± 0.399	0.718
rs2312147	*VRK2*	0.109 ± 0.434	0.803	0.158 ± 0.442	0.721	0.235 ± 0.413	0.570
rs7561688	*VRK2*	0.749 ± 0.436	0.090 *	0.542 ± 0.449	0.231	0.427 ± 0.418	0.310
rs986512	*VRK2*	−0.346 ± 0.422	0.414	−0.019 ± 0.434	0.964	−0.403 ± 0.398	0.314
rs1051061	*VRK2*	−0.060 ± 0.462	0.897	0.057 ± 0.470	0.904	−0.228 ± 0.437	0.604
rs848292	*VRK2*	0.429 ± 0.462	0.356	−0.057 ± 0.474	0.904	0.391 ± 0.440	0.377
rs848291	*VRK2*	0.135 ± 0.453	0.766	0.002 ± 0.461	0.997	0.314 ± 0.427	0.465
rs10830963	*MNTR1B*	−0.400 ± 0.485	0.412	−0.586 ± 0.489	0.235	−1.117 ± 0.430	0.011
Specific PRS	PRS	0.516 ± 0.135	<0.001	0.867 ± 0.248	0.001	0.508 ± 0.127	<0.001

Values are presented as β ± SE (standard error); SNP, single nucleotide polymorphism; PRS, polygenic risk score; *p*-values were obtained in multivariable linear regressions models adjusted for age, sex, and kcal.

## Data Availability

Neither the participants’ consent forms nor ethics approval included permission for open access. However, we follow a controlled data-sharing collaboration model, and data for collaborations will be available upon request pending application and approval. Investigators who are interested in this study can contact the corresponding author Dolores Corella (dolores.corella@uv.es).

## Supplementary Materials

The following supporting information can be downloaded at: https://www.mdpi.com/article/10.3390/medicina58091294/s1, Table S1: Association between lifestyle and clinical parameters with clock gene expression.
