# Supplementary material for: Influence of DNA-Polymorphisms in Selected Circadian Clock Genes on Clock Gene Expression in Subjects from the General Population and Their Association with Sleep Duration
Source: Medicina (Kaunas). 2022 Sep 16;58(9):1294. doi: 10.3390/medicina58091294 (PMC9506325; doi:10.3390/medicina58091294)
Supplement: Supplementary file 1 [file medicina-58-01294-s001.zip › medicina-1869569-supplementary.pdf]

medicina

Influence of DNA-Polymorphisms in Selected Circadian Clock Genes on Clock Gene Expression in Subjects from the General Population and Their Association with Sleep Duration

Rocío Barragán, José V. Sorlí, Oscar Coltell, Inmaculada Gonzalez-Monje, Rebeca Fernández-Carrión, Laura V. Villamil, Olga Portolés, Dolores Corella, Carolina Ortega-Azorín and Eva M. Asensio

SUPPLEMENTARY MATERIAL

Table S1. Association between lifestyle and clinical parameters with clock gene expression.

	**CLOCK expression (β ± SD; p-value)**	**PER1 expression (β ± SD; p-value)**	**VRK2 expression (β ± SD; p-value)**
Age	-0.28 ± 0.02; 0.111	0.00 ± 0.02; 0.897	-0.01 ± 0.02; 0.587
Obesity	-0.21 ± 0.65; 0.748	0.21 ± 0.66; 0.754	-0.10 ± 0.60; 0.864
Diabetes	1.19 ± 1.30; 0.360	1.02 ± 1.30; 0.435	1.34 ± 1.19; 0.263
MedDiet	0.01 ± 0.13; 0.971	-0.07 ± 0.13; 0.570	0.05 ± 0.12; 0.675
Sleep duration	0.08 ± 0.35; 0.815	0.26 ± 0.34; 0.450	-0.14 ± 0.33; 0.676
Waketime	-0.11 ± 0.32; 0.724	0.17 ± 0.31; 0.599	-0.18 ± 0.30; 0.545
Smoking	-1.10; 0.79; 0.165	-0.26 ± 0.78; 0.741	-1.30 ± 0.70; 0.067

Values are represented as beta-values (β) and standard deviation (SD). Multivariable linear regressions adjusted for age and sex. Age, MedDiet, Sleep duration and waketime was tested as a continuous variables. Obesity, diabetes and smoking was testes as a categorical variable. MedDiet: Adherence to the Mediterranean Diet.
